# Clinical management of hip fractures in elderly patients with dementia and postoperative 30‐day mortality: A population‐based cohort study

**DOI:** 10.1002/brb3.1823

**Published:** 2020-09-06

**Authors:** Jindong Ding Petersen, Volkert Dirk Siersma, Sonja Wehberg, Connie Thurøe Nielsen, Bjarke Viberg, Frans Boch Waldorff

**Affiliations:** ^1^ Research Unit for General Practice Department of Public Heath University of Southern Denmark Odense Denmark; ^2^ Department of Mental Health Vejle Mental Health Services in the Region of Southern Denmark Vejle Denmark; ^3^ Research Unit for General Practice and Section of General Practice Department of Public Health University of Copenhagen Copenhagen Denmark; ^4^ Department of Orthopaedic Surgery and Traumatology Kolding Hospital – Part of Hospital Lillebælt Kolding Denmark

**Keywords:** 30‐day mortality, clinical management, dementia, hip fracture surgery

## Abstract

**Objectives:**

Patients with dementia have an increased 30‐day mortality after hip fracture. We investigated clinical management including time to surgery, out‐of‐hours admission and surgery, surgery on weekends, surgery volume per ward, and anesthesia technique for this excess mortality risk.

**Method:**

This register‐ and population‐based study comprised 12,309 older adults (age 70+) admitted to hospital for a first‐time hip fracture in 2013–2014, of whom 11,318 underwent hip fracture surgery. Cox proportional hazards regression models were applied for the analysis.

**Results:**

The overall postoperative 30‐day mortality was 11.4%. Patients with dementia had a 1.5 times increased mortality risk than those without (HR = 1.50 [95% CI 1.31–1.72]). We observed no time‐to‐surgery difference by patient dementia status; additionally, the excess mortality risk in patients with dementia could not be explained by the clinical management factors we examined.

**Conclusions:**

Increased mortality in patients with dementia could not be explained by the measured preoperative clinical management. Suboptimal handling of postoperative complication and rehabilitation are to be investigated for their role in the witnessed increased mortality for patients with dementia.

## Graphical abstract

In this register‐ and population‐based cohort study of 12,309 older adults (age 70+) with hospital admission for a first‐time hip fracture in 2013–2014, we found an increased 30‐day postoperative mortality in patients with dementia. However, this excess mortality was not explained by the clinical factors examined (out‐of‐hours admission, surgery on weekends, anesthesia technique, and surgery volume per ward). Additionally, we found no difference in time to surgery by the patient's dementia status.

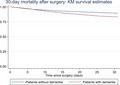

**Key points**

- Patients with dementia were at a higher risk of dying within 30 days after hip fracture surgery.
- This excess postoperative 30‐day mortality in patients with dementia could not be explained by the measured preoperative clinical management.

## 1 INTRODUCTION

Hip fractures are common among the elderly, with substantial consequences of decreased mobility and increased morbidity and mortality (de Luise, Brimacombe, Pedersen, & Sorensen, 2008). Approximately 20%–30% of elderly with hip fractures die within 1 year and 10%–15% die within 30 days (Hu, Jiang, Shen, Tang, & Wang, 2012; Jantzen, Madsen, Lauritzen, & Jorgensen, 2018; Mundi, Pindiprolu, Simunovic, & Bhandari, 2014). As the population ages, the rapid increase in hip fractures poses a major public health concern and clinical challenge for society at large (Cooper, Campion, & Melton, 1992).

People with dementia are up to three times more likely than their cognitively intact counterparts to experience a hip fracture, often via accidents including falls (Friedman, Menzies, Bukata, Mendelson, & Kates, 2010; Petersen et al., 2018). After hip fracture, patients with dementia have a higher risk of dying within 30 days after surgical treatment compared with other hip fracture patients (Bai et al., 2018; Liu, Wang, & Xiao, 2018), and the reasons for this are not yet fully understood. Although this higher mortality may be linked to the combined effects of dementia, comorbidities, and intake of multiple medications Smith, Pelpola, Ball, Ong, and Myint (2014), clinical management of hip fracture surgery in hospital settings may play a role.

Studies among general hip fracture patients have revealed that time to surgery (TTS) is a risk factor for postoperative mortality (Chang et al., 2018; Klestil et al., 2018). Other clinical management factors such as out‐of‐hours admission and surgery, surgery unit volume, and anesthesia technique have been shown as risk factors for postoperative mortality, but some of these results are contradictory (Daugaard et al., 2012; Foss & Kehlet, 2006; Guay, Parker, Gajendragadkar, & Kopp, 2016; Hagino et al., 2015; Kristensen, Thillemann, & Johnsen, 2014; Malik, Panni, Masri, & Noordin, 2018; Simunovic et al., 2010; Wiegers et al., 2019; Zhou et al., 2016). Studies of these clinical management factors in hip fracture patients with dementia for mortality risk are limited and have been conducted in different healthcare systems and are heterogenic in study methodology Yonezawa, Yamazaki, Atsumi, and Obara (2009); Petersen, Jorgensen, Hansen, & Duus, 2006; Johansen et al., 2017).

In Denmark, national clinical guidelines for hip fracture patient care are implemented in routine daily clinical practice, presumably regardless of patients' dementia status, but in real life, discrepancies may exist. Hence, this setting was apt for our study to investigate whether differences in the abovementioned clinical management factors may explain the excess postoperative 30‐day mortality risk for hip fracture patients with dementia.

## 2 STUDY METHODS

### 2.1 Study design and population

This was a population‐based study using data from Danish national registers linked using the unique civil registration (CPR) number assigned to each individual at birth and to persons who hold a Danish residence permit upon immigration (Pedersen, 2011).

The study population was all residents in Denmark aged 70 or older (70+) who had a first‐time hip fracture admission to a hospital in Denmark between January 1, 2013, and December 31, 2014 (2013–2014).

**FIGURE 1:**
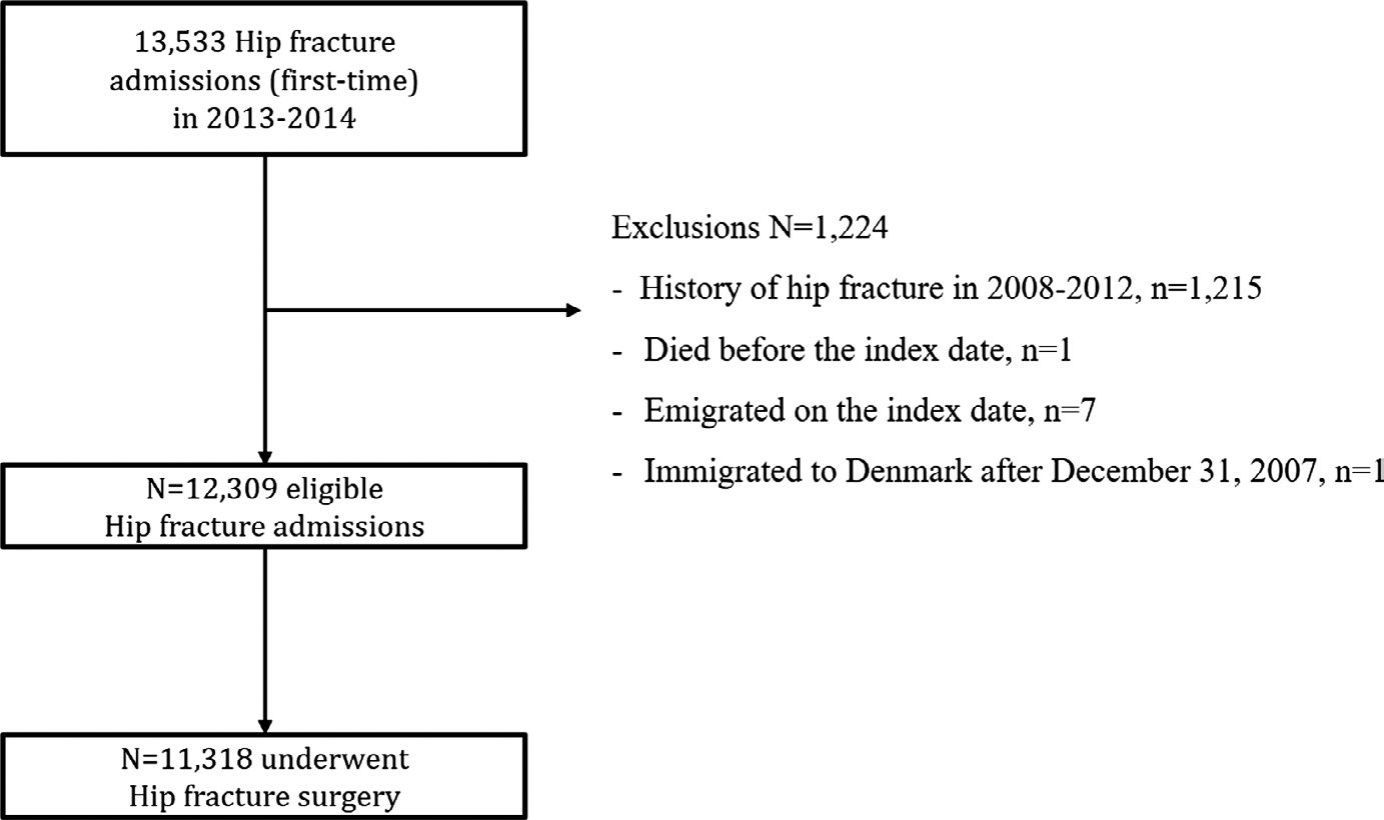
Flowchart of the study participant selection

We excluded individuals from the study if they met any of the following criteria: (a) history of hip fracture in 2008–2012, (b) died before the index date of hip fracture admission, and (c) emigration on the index date. In addition, we also excluded individuals who immigrated to Denmark later than December 31, 2007, due to potentially incomplete information on hip fracture, chronic diseases, and medication in the registers for the period of 2008 to 2012 (Figure 1).

Records of hip fracture and hip fracture surgery were extracted from the Danish National Patient Register (DNPR) (Lynge, Sandegaard, & Rebolj, 2011). The date of hospital admission for a first‐time hip fracture in 2013–2014 was defined as the index date. The eligible participants were followed for 30 days from both the date of the hip fracture admission (*n* = 12,309) and the date of the hip fracture surgery (*n* = 11,318) for mortality assessment.

International Classification of Diseases, 10th Revision (ICD‐10) codes were used to define hip fractures as fracture of femoral head or neck (S720), pertrochanteric fracture (S721), or subtrochanteric fracture (S722) in 2013–2014 from the DNPR. Additionally, ICD‐10 code M809B for osteoporotic hip fracture was used together with hip fracture codes (S72*) for inclusion. Using the same hip fracture diagnosis codes, an individual's history of hip fracture records in 2008–2012 was also traced in the register for exclusion purpose. Both primary and secondary diagnoses were included with any type of inpatient, outpatient, or emergency department registration from Danish hospitals. The Nordic Medico‐Statistical Committee codes for arthroplasty (KNFB*) and osteosynthesis (KNFJ*) were used to extract potential hip fracture surgery performed on eligible study participants.

### 2.2 Variables of interests

#### 2.2.1 30‐day mortality

We defined 30‐day postoperative mortality as death within the first 30 days after hip fracture surgery (in or out of hospital, all‐cause). We obtained information on death (all‐cause) including the date of death from the CPR Pedersen, (2011), which records an individual's vital status and date of death.

#### 2.2.2 Dementia assessment

A person was identified with dementia (any type) if they fulfilled one of the following criteria: (1) a primary or secondary dementia diagnosis (ICD‐10 codes: F00‐03, G30, G318E) in the DNPR or in the Danish Psychiatric Central Research Register (PCRR) (Mors, Perto, & Mortensen, 2011), and/or (2) at least one prescribed antidementia drug registration in the Danish National Prescription Registry (LMDB) (Pottegard et al., 2017).

Anatomical Therapeutic Chemical (ATC) codes were used to identify dementia‐related medications prescribed in Denmark (ATC codes: N06DA01, N06DA02, N06DA03, N06DA04, N06DX01) (Pottegard et al., 2017; Schmidt et al., 2015). The prescription registrations were used in this study in order to capture patients with dementia but missing a diagnosis in the DNPR or PCRR.

We traced the history of an individual's dementia diagnosis and dementia‐related medications for the period of 2008–2014. For this study, the identified date of dementia had to be at least one day before the index date of the hip fracture hospital admission registered in 2013–2014.

#### 2.2.3 Medical comorbidity

We used the seven most common chronic diseases present in the Danish elderly population for assessing medical comorbidity (Moth, Vestergaard, & Vedsted, 2012): type 2 diabetes (T2D) (ICD‐10 code: E11), chronic obstructive pulmonary disease (COPD) (ICD‐10 codes: J40–J44), ischemic heart disease (IHD) (ICD‐10 codes: I20–I25), depression (ICD‐10 codes: F32–F33), hypertension (ID‐10 codes: I10, I15), stroke (ICD‐10 codes: I60–I69), and atrial fibrillation (AF) (ICD‐10 code: I48), which were extracted from the DNPR for the period of 2008–2014.

We grouped these diseases into four categories: 0, 1, 2, or 3 or more (3+) comorbid diseases, in any combination, based on the number of diseases present on the index date of hospital admission for hip fracture, as well as on the date of the hip fracture surgery.

#### 2.2.4 Sedative medication

A person was defined as taking sedative medication if they had taken any medication of four types registered in the LMDB: sleeping medications (ATC code: N05C), antipsychotics (ATC code: N05A), antianxiety medications (ATC code: N05B), and antidepressants (ATC code: N06A), within 100 days prior to the index date for hip fracture admission (The largest package of prescribed sleeping medication in Denmark contains 100 pills, and the usual dose is 1 pill a day). We grouped usage of these medications into three categories: 0, 1, 2, or 3 and more (3+), in any combination.

#### 2.2.5 Clinical management factors in hip fracture surgery

##### Time to surgery

We defined TTS as the time from the hospital admission for hip fracture to the time hip fracture surgery was performed and measured in hours. There were a few cases with a zero‐time registration between admission and surgery. If the two events occurred on the same day, then we set the TTS equal to 0.5 days, that is, 12 hrs.

##### Out‐of‐hours admission and surgery

We defined out of hours as any admission and surgery in the time period of 16:01–07:59.

##### Surgery on weekend

Any surgery performed on Saturdays, Sundays, and on Danish public holidays in 2013–2014 was defined as surgery on weekends.

##### Anesthesia technique

Anesthesia technique used for hip fracture surgery was divided into two groups: general anesthesia (NAAC*), and regional anesthesia (NAAD*) includes both spinal (NAAD1*) and epidural anesthesia (NAAD0*). Since very few patients were operated on with local anesthetic and/or conscious sedation (NAAB*), we categorized them with the regional anesthesia group.

##### Surgery volume per ward

Surgery volume per ward (identified using the six‐digit code for each hospital) was measured by the total number of registered hip fracture surgeries performed (only arthroplasty (KNFB*) and osteosynthesis (KNFJ*) surgeries were included) in 2013–2014. We only counted one procedure in cases where several procedures were recorded for the same patient in the study period.

Based on the distribution [median 233 (25th‐75th percentile 203–699)] per unit or ward, we grouped the volume of surgeries performed into three groups: (1) low volume (fewer than 200 surgeries), (2) medium volume (200 to 700 surgeries), and high volume (more than 700 surgeries).

#### 2.2.6 Other covariates

The study participants' age, sex, marital status, immigration, and emigration status were all extracted from the CPR Pedersen, (2011). The participants' education information was extracted from the Population Education Register (Jensen & Rasmussen, 2011).

The patients were divided into three age groups: 70–79, 80–90, or 90 years and older (90+). Marital status was grouped into four categories: married (including partnership or cohabitation), divorced (including dissolved partnership), widowed (including partner died), or single (no cohabitation). Education level was grouped according to the individual's highest education level in years: low education (<10 years), medium education (10–12 years), or higher education (>12 years).

We also included American Society of Anesthesiologists Physical Status Classification System codes (ASA class) scale as a covariate (Daabiss, 2011). Given that ASA class scale of 5 indicates a patient who is not expected to survive 24 hr with or without surgery for the present study, we included a scale of 1–4 to group patients: A lower number indicates patients who are more fit for surgery, while a higher number indicates patients less fit for surgery. In the DNPR, ASA class is recorded using codes EZA*.

### 2.3 Statistical analysis

We described the age group, sex, education, marital status, presence of comorbid diseases, and use of sedative medication by the index date of hospital admission for hip fracture in patients with and without dementia. We also described the characteristics of hip fracture surgeries in 2013–2014, including clinical management for hip fracture surgery by dementia status.

The 30‐day postsurgery mortality was time to event. Patients were censored at the end of the study or at their date of emigration. However, for the TTS, patients were also censored if they died before the hip fracture surgery. We applied Cox proportional hazards regression (Cox regression) to estimate association between dementia and the study outcomes.

For the association between dementia and TTS, the multivariable Cox regression was adjusted for age group, sex, education, marital status, comorbid diseases, sedative medications, and out‐of‐hours hospital admissions for hip fracture.

For the association between dementia and 30‐day postsurgery mortality, we applied three Cox regression models. Model A was univariable analysis of dementia for 30‐day mortality after hip fracture surgery. Model B was Model A adjusted for confounders (age group, sex, education, marital status, comorbidity, and intake of sedative medications). Model C was Model B adjusted for clinical management factors including out‐of‐hours admissions, out‐of‐hours surgery, surgery on weekends, anesthesia technique, and surgery volume per ward. Additionally, ASA class scores, type of surgery, and type of hip fracture were all adjusted for in Model C.

The risk estimations are presented as hazard ratios (HRs) with corresponding 95% confidence intervals (95% CIs). All statistical analysis was performed using STATA version 14.0 (StataCorp LP).

### 2.4 Ethical consideration

Complying with European data protection rules, the Danish Data Protection Agency registered the project (Journal no. 2016‐41‐4674). According to Danish law, review by an ethics board or patient consent is not required for purely register‐based studies.

## 3 RESULTS

### 3.1 Population characteristics at hospital admission for hip fracture

A total of 12,309 first‐time hip fracture hospital admissions were identified in the period 2013–2014 among older people (age 70+) in Denmark, and among them, 1,853 (15.1%) patients had a diagnosis of dementia. There were a higher proportion of older, female, and divorced or widowed patients, and more patients with comorbid chronic diseases and taking sedative medications in the dementia group than in the nondementia group (Table 1). More than half (53.6%) of hip fracture patients were admitted out‐of‐hours, but no significant difference by dementia status was found.

**Table 1:** Characteristics of patients with hospital admission for first‐time hip fracture in 2013–2014 in Denmark

Variables *N* (%)	All	Patients without dementia	Patients with dementia
	12,309 (100.0)	10,456 (100.0)	1853 (100.0)
Days since dementia diagnosis			
1+			351 (18.9)
365+			571 (30.8)
1,095 (3 years)+			931 (50.2)
Age, median (range)	84 (79–89)	84 (78–89)	86 (81–89)
Age group			
70–79	3,484 (28.3)	3,132 (30.0)	352 (19.0)
80–89	6,010 (48.8)	4,953 (47.4)	1,057 (57.0)
90+	2,815 (22.9)	2,371 (22.7)	444 (24.0)
Sex			
Male	3,491 (28.4)	3,049 (29.2)	442 (23.9)
Female	8,818 (71.6)	7,407 (70.8)	1,411 (76.1)
Marital status			
Married/cohabiting	3,580 (29.1)	3,077 (29.4)	503 (27.1)
Divorced	1,276 (10.4)	1,070 (10.2)	206 (11.1)
Widowed	6,771 (55.0)	5,699 (54.5)	1,072 (57.9)
Never married	682 (5.5)	610 (5.8)	72 (3.9)
Education			
<10 years	6,482 (52.7)	5,469 (52.3)	1,013 (54.7)
10–12 years	3,047 (24.7)	2,583 (24.7)	454 (24.5)
>12 years	1,349 (11.0)	1,164 (11.1)	185 (10.0)
Missing	1,441 (11.7)	1,240 (11.9)	201 (10.8)
Chronic diseases			
T2D	599 (4.9)	519 (5.0)	80 (4.3)
COPD	3,506 (28.5)	3,053 (29.2)	453 (24.4)
IHD	1923 (15.6)	1662 (15.9)	261 (14.1)
Depression	5,525 (44.9)	4,271 (40.8)	1,254 (67.7)
Hypertension	1,177 (9.6)	1,036 (9.9)	141 (7.6)
Stroke	1788 (14.5)	1527 (14.6)	261 (14.1)
AF	1,478 (12.0)	1,257 (12.0)	221 (11.9)
Number of comorbid diseases^a^			
0	3,260 (26.5)	2,938 (28.1)	322 (17.4)
1	4,422 (35.9)	3,664 (35.0)	758 (40.9)
2	2,904 (23.6)	2,405 (23.0)	499 (26.9)
≥3	1723 (14.0)	1,449 (13.9)	274 (14.8)
Sedative medication use			
Sleeping medications	1631 (13.3)	1,458 (13.9)	173 (9.3)
Antipsychotics	809 (6.6)	480 (4.6)	329 (17.8)
Antianxiety medications	973 (7.9)	833 (8.0)	140 (7.6)
Antidepressants	3,377 (27.4)	2,423 (23.2)	954 (51.5)
Quantity of sedative medication^b^ use			
0	7,283 (59.2)	6,578 (62.9)	705 (38.0)
1	3,572 (29.0)	2,778 (26.6)	794 (42.8)
2	1,173 (9.5)	901 (8.6)	272 (14.7)
≥3	281 (2.3)	199 (1.9)	82 (4.4)
Out‐of‐hours admission^c^ (16–07)	6,594 (53.6)	5,573 (53.3)	1,021 (55.1)

Abbreviations: AF, atrial fibrillation; COPD, chronic obstructive pulmonary disease; IHD, ischemic heart disease; T2D, type 2 diabetes.

^a^ Any combination of the chronic diseases including T2D, COPD, IHD, depression, hypertension, stroke, and AF.

^b^ Any combination of sleeping medication, antipsychotics, antianxiety medications, and antidepressants.

^c^ Hospital admission for a first‐time hip fracture in 2013–2014.

### 3.2 Dementia and time to surgery (TTS)

Among 12,309 hospital admissions for a first‐time hip fracture in 2013–2014, 11,318 patients underwent hip fracture surgery, with a median (and 25th‐75th percentile) TTS of 22 hr (15–31). Patients with dementia experienced a median TTS of 21 hr (15–31), whereas patients without dementia have a median TTS of 23 hr (15–35).

However, multivariable analysis adjusted for age group, sex, marital status, education, comorbid diseases, sedative medications, and out‐of‐hours hospital admissions for hip fracture showed no significant TTS difference (HR = 1.04 [95% CI = 0.99–1.10]) between patients by dementia status. Additionally, age, sex, marital status, education, comorbid diseases, and sedative medication use showed no effect on TTS (Table 2).

**Table 2:** Time to surgery after hip fracture hospital admissions (*n* = 12,309)

Risk factors	HR (95% CIs)	
	Univariable	Multivariable
Dementia (yes)	1.06 (1.01–1.12)	1.04 (0.99–1.10)
Days since dementia diagnosis		
No dementia	Ref	
1+	1.05 (0.94–1.18)	
365+	1.03 (0.95–1.13)	
1,095 (3 years)+	1.08 (1.00–1.16)	
Age group		
70–79	Ref	Ref
80–89	1.04 (0.99–1.08)	1.03 (0.98–1.08)
90+	1.05 (1.00–1.11)	1.05 (0.99–1.12)
Sex		
Female	Ref	Ref
Male	0.90 (0.87–0.94)	0.92 (0.88–0.96)
Civil status		
Married/cohabiting	Ref	Ref
Divorced	1.05 (0.98–1.12)	1.04 (0.97–1.11)
Widowed	1.03 (0.99–1.08)	0.99 (0.95–1.04)
Never married	1.04 (0.96–1.14)	1.03 (0.94–1.12)
Education		
<10 years	Ref	Ref
10–12 years	0.96 (0.91–1.00)	0.98 (0.93–1.02)
>12 years	0.95 (0.89–1.01)	0.96 (0.90–1.02)
Missing	0.97 (0.92–1.03)	0.95 (0.88–1.02)
Number of comorbid diseases^a^		
0	Ref	Ref
1	1.01 (0.96–1.06)	0.99 (0.95–1.04)
2	0.94 (0.89–0.99)	0.92 (0.87–0.98)
≥3	0.88 (0.83–0.94)	0.87 (0.81–0.93)
Quantity of sedative medication use^b^		
0	Ref	Ref
1	1.01 (0.97–1.05)	1.02 (0.98–1.07)
2	0.99 (0.93–1.05)	1.01 (0.94–1.08)
≥3	0.98 (0.86–1.11)	1.00 (0.88–1.14)
Out‐of‐hours admissions (16–07)^c^	1.17 (1.13–1.21)	1.17 (1.13–1.22)

Abbreviations: 95% CIs, 95% confidence intervals; HR, hazard ratio.

^a^ Any combination of chronic diseases including type 2 diabetes, chronic obstructive pulmonary disease, ischemic heart disease, depression, hypertension, stroke, and atrial fibrillation.

^b^ Any combination of sleeping medications, antipsychotics, antianxiety medications, and antidepressants.

^c^ Hospital admission for hip fracture surgery.

### 3.3 Characteristics of hip fracture surgeries

Among the 11,318 patients who underwent a hip fracture surgery, half (51.7%) had a fracture of the femoral head or neck; nearly two‐thirds of the total study population received osteosynthesis surgery; and more than one‐thirds (37.8%) of the total surgeries were performed out of normal business hours, and 29.4% on weekends (Table 3).

**Table 3:** Characteristics of 11,318 hip fracture surgeries by dementia status

Variables *N* (%)	All	Without dementia	With dementia
Hip fracture surgery	11,318 (100.0)	9,611 (100.0)	1707 (100.0)
Out‐of‐hours admission	6,132 (54.2)	5,181 (53.9)	951 (55.7)
Out‐of‐hours surgery (16–07)	4,274 (37.8)	3,573 (37.2)	701 (41.1)
Surgery on weekends (yes)	3,323 (29.4)	2,795 (29.1)	528 (30.9)
Anesthesia technique			
General	1784 (15.8)	1,496 (15.6)	288 (16.9)
Regional	2,555 (22.6)	2,214 (23.0)	341 (20.0)
No information	6,979 (61.7)	5,901 (61.4)	1,078 (63.2)
Surgery volume per ward in 2013–2014			
Low (1–199)	376 (3.3)	319 (3.3)	57 (3.3)
Middle (200–699)	3,350 (29.6)	2,859 (29.7)	491 (28.8)
High (700+)	7,592 (67.1)	6,433 (66.9)	1,159 (67.9)
Type of hip fracture			
Femur head and neck	5,855 (51.7)	4,937 (51.4)	918 (53.8)
Pertrochanteric	4,425 (39.1)	3,764 (39.2)	661 (38.7)
Subtrochanteric	793 (7.0)	687 (7.1)	106 (6.2)
Other	245 (2.2)	223 (2.3)	22 (1.3)
Type of surgery			
Arthroplasty	4,111 (36.3)	3,509 (36.5)	602 (35.3)
Osteosynthesis	7,207 (63.7)	6,102 (63.5)	1,105 (64.7)
ASA class^a^			
1–2	1,053 (9.3)	943 (9.8)	110 (6.4)
3	1,073 (9.5)	818 (8.5)	255 (14.9)
4	106 (0.9)	84 (0.9)	22 (1.3)
Missing	9,086 (80.3)	7,766 (80.8)	1,320 (77.3)

^a^ American Society of Anesthesiologists Physical Status Classification System score.

There was no significant difference in clinical management of hip fracture surgery, including out‐of‐hours admission, surgery on weekends, and surgery volume per ward, between patients with and without dementia. However, patients with dementia were more likely to undergo an operation out of hours and receive general anesthesia than those without dementia (*p* < .05).

### 3.4 Dementia and postoperative 30‐day mortality

**FIGURE 2:**
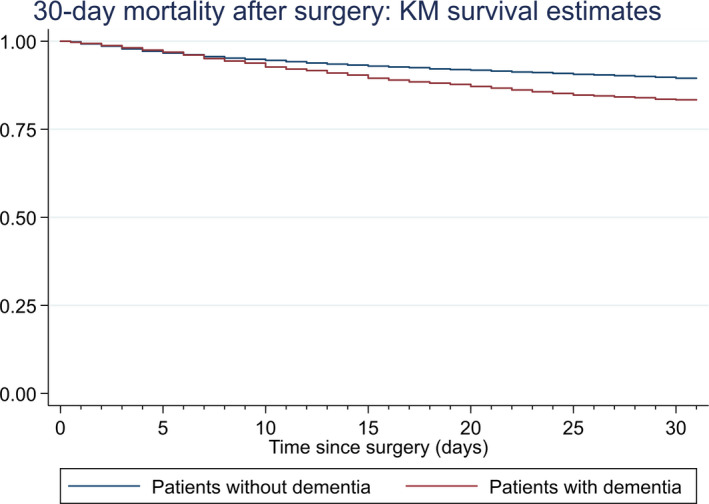
Kaplan–Meier (KM) estimate for 30‐day postsurgery mortality by dementia status

Of 11,318 patients who underwent hip fracture surgery, 1,292 (11.4%) died within 30 days after the surgery, including 284 patients with dementia. The computed Kaplan–Meier estimate of postoperative 30‐day mortality showed a significant higher mortality in patients with dementia (Figure 2) with about 1.6‐fold increased hazard rate revealed in the crude Cox regression analysis compared with patients without dementia (HR = 1.62 [95% 1.42–1.85]).

In multivariable analysis adjusted for confounders including age group, sex, education, marital status, comorbidities, and sedative medications, patients with dementia had a risk of dying within 30 days after hip fracture surgery that was 1.5 times higher than the risk for patients without dementia (HR = 1.50 [95% CI 1.31–1.72]) (Table 4).

**Table 4:** Multivariable Cox regression for dementia and 30‐day mortality after hip fracture surgery

Variables	HR (95% CIs)	
	Multivariable 1^a^	Multivariable2^b^
Dementia (yes)	1.50 (1.31–1.72)	1.50 (1.31–1.72)
Age group		
70–79	Ref	Ref
80–89	1.92 (1.62–2.28)	1.90 (1.60–2.25)
90+	3.45 (2.84–4.19)	3.38 (2.78–4.11)
Male	2.33 (2.06–2.63)	2.31 (2.04–2.61)
Marital status		
Married/cohabiting	Ref	Ref
Divorced	1.13 (0.91–1.40)	1.12 (0.90–1.39)
Widowed	1.30 (1.13–1.51)	1.29 (1.12–1.50)
Never married	1.01 (0.76–1.33)	1.01 (0.76–1.34)
Education		
<10 years	Ref	Ref
10–12 years	0.92 (0.80–1.06)	0.92 (0.80–1.07)
>12 years	0.89 (0.72–1.09)	0.89 (0.72–1.09)
Missing	1.33 (1.12–1.57)	1.33 (1.12–1.57)
Quantity of comorbid diseases^c^		
0	Ref	Ref
1	1.21 (1.03–1.41)	1.20 (1.02–1.40)
2	1.43 (1.20–1.69)	1.40 (1.18–1.66)
≥3	1.65 (1.37–1.99)	1.61 (1.33–1.95)
Quantity of sedative medications^d^		
0	Ref	Ref
1	1.18 (1.04–1.34)	1.17 (1.03–1.33)
2	1.11 (0.92–1.34)	1.11 (0.91–1.34)
≥3	1.68 (1.23–2.29)	1.60 (1.17–2.18)
Out‐of‐hours admission		1.06 (0.95–1.19)
Out‐of‐hours surgery (16–07)		0.97 (0.87–1.09)
Surgery on weekends (yes)		0.97 (0.86–1.09)
Anesthesia technique		
General		Ref
Regional		0.82 (0.69–0.99)
No information		0.89 (0.76–1.04)
Surgery volume per ward in 2013–2014		
Low (1–199)		Ref
Medium (200–699)		1.10 (0.77–1.55)
High (700+)		1.26 (0.90–1.76)
Type of surgery		
Arthroplasty		Ref
Osteosynthesis		0.78 (0.66–0.92)
Type of hip fracture		
Femur head or neck		Ref
Pertrochanteric		1.25 (1.06–1.47)
Subtrochanteric		1.34 (1.04–1.71)
Other		1.25 (0.85–1.83)
ASA class		
1–2		Ref
3		1.32 (1.01–1.73)
4		3.15 (2.11–4.71)
Missing		1.29 (1.03–1.63)

Abbreviations: 95% CIs = 95% confidence intervals; HR, hazard ratio.

^a^ Model 1 adjusted for age group, sex, education, marital status, comorbid diseases, and sedative medications.

^b^ Model 2 = Model 1 adjusted for all other variables listed in the table.

^c^ Any combination of chronic diseases including type 2 diabetes, chronic obstructive pulmonary disease, ischemic heart disease, depression, hypertension, stroke, and atrial fibrillation.

^d^ Any combination of sleeping medications, antipsychotics, antianxiety medications, and antidepressants.

Further adjusting for clinical management factors for hip fracture surgery, including out‐of‐hours admission, out‐of‐hours surgery, surgery on weekends, anesthesia technique, and surgery volume per ward, did not change the estimate for excess mortality for dementia patients (HR = 1.50 [95% CI 1.31–1.72]). However, patients who received regional anesthesia showed an 18% reduction of 30‐day postsurgery mortality risk compared with the patients who received general anesthesia.

Increased age, male sex, comorbid diseases, sedative medications, and higher ASA scores were significantly associated with 30‐day postsurgery mortality.

A sensitivity analysis the using Charlson Comorbidity Index (Charlson, Pompei, Ales, & MacKenzie, 1987) instead of the comorbidity we defined did not change the estimates for mortality risk for the other factors.

## 4 DISCUSSION

Among the elderly individuals (age 70+) admitted to hospital for a first‐time hip fracture in 2013–2014 who underwent hip fracture surgery, patients with dementia had a 1.5 times (HR = 1.50 [95% CI 1.31–1.72]) higher postoperative 30‐day mortality compared with patients without dementia. However, we observed no TTS difference by patient dementia status; additionally, the excess mortality risk in patients with dementia could not be explained by the clinical management factors we examined.

### 4.1 Comparison with existing studies

To the best of our knowledge, our study is the first population‐based study to investigate dementia for postoperative 30‐day mortality in Denmark by taking into account the clinical management factors investigated in this study. Other studies, although in line with our postoperative mortality risk estimations in patients with dementia, lack clinical management factors for comparison (Bai et al., 2018; Pedersen, Ehrenstein, & Szepligeti, 2017).

Managing patients with dementia has great challenges in clinical practice Krupic, Eisler, Skoldenberg, and Fatahi (2016). Due to the nature of the disease, communication with dementia patients and recognizing their wants and needs can be difficult (Judd, 2017; Tarazona‐Santabalbina et al., 2015). It often requires of staff extra time and specialized knowledge of dementia Klestil et al., (2018). These requirements may be difficult to meet in surgery departments, for example, due to heavy workloads and an inadequate supply of personnel with expertise in dementia care, and this situation may worsen as the population ages (Simunovic et al., 2010). Furthermore, patients undergoing hip fracture surgery include individuals with dementia at all stages of severity. Among those with moderate‐to‐severe dementia, nearly 30% to 90% of them manifest behavioral and psychological symptoms, and such symptoms may play a modifying role with hip fracture for clinical management (Muller‐Spahn, 2003). All these factors mentioned previously may lead to low priority in clinical practice. However, we found no significant difference in these clinical management factors measured by patient dementia status, which reflects that professionals in the Danish health care system treat hip fracture patients fairly, regardless of dementia diagnosis.

In our study, out‐of‐hours admission, out‐of‐hours surgery, and surgery on weekends showed no significant influence on the excess postoperative 30‐day mortality. A study by Kristiansen et al in general hip fracture patients reported that patients admitted to hospitals during weekends in 2010–2013 had a 13% higher 30‐day mortality than those admitted during weekdays (Kristiansen, Kristensen, Norgard, Mainz, & Johnsen, 2016). We studied hip fractures that occurred in 2013–2014: Improvements in hospital care of hip fractures in the intervening years may explain the inconsistent results between Kristiansen et al and our study.

Furthermore, we found that among all patients whose anesthesia technique was recorded in the DNPR, those who received regional anesthesia had an 18% reduction in 30‐day postsurgery mortality compared with general anesthesia. A systematic review and meta‐analysis by Guay et al published by the Cochrane Library in 2016 examined 11 studies and concluded that there was no difference in mortality at 30 days between these two techniques, but they also concluded that the low quality of the evidence calls for more randomized clinical trial contributions (Guay et al., 2016). It should be noted that in our study population, among patients who underwent hip fracture surgery, 61.7% of their records lacked anesthesia information; however, this missing information showed no effect on postoperative 30‐day mortality.

Our results revealed that being older, male, comorbid with more chronic diseases, taking more sedative medications, and having a higher ASA class score were more important prognostic factors for 30‐day mortality than the clinical management aspects we assessed. These factors are often present in patients with dementia. Therefore, when admitting a hip fracture patient, proper screening should take place to identify these prognostic factors and guide extra attention to mitigation of same: Cautious care may improve survival rate in this population.

### 4.2 Study strengths and limitations

Our study has a number of important strengths. In this population‐based cohort study, all variable data were extracted from Danish national registers. The CPR number‐linked information tracked from the registers resulted in no loss to follow‐up within our cohorts and minimized selection bias.

The limitations of this study are the focus on certain pre‐ and perioperative clinical management factors. While we feel that we have addressed adequately the major pre‐ and perioperative factors that have been shown to influence on postoperative mortality, the study does not address postoperative complications and care. Suboptimal compliance to rehabilitation regimes for patients with dementia may explain the excess mortality and something that can be improved upon (Seitz, Gill, & Austin, 2016). Also, delirium, which often accompanies dementia, may explain part of the excess mortality (Mosk et al., 2017). Further studies should focus on the role of postoperative clinical management in the excess mortality after surgery for dementia patients, and these studies should not be purely register‐based.

Severity of dementia is linked to functional recovery and mortality after hip fracture (Tarazona‐Santabalbina et al., 2015). Given that the DNPR lacks validation of dementia severity registration (Phung et al., 2007), we adjusted using the number of days since identification of dementia as a proxy, and found it had no influence on the 30‐day mortality. With continuing development of the Danish Clinical Registries for dementia (The Danish Clinical Registries (RKKP), 2018), studies of postoperative clinical management aspects and dementia severity for relative health outcomes may be possible in the future.

## 5 CONCLUSION

Among elderly patients with a first‐time hip fracture in 2013–2014, we found an increased 30‐day mortality in patients with dementia. However, the increased postoperative 30‐day mortality was not explained by the surveyed clinical factors. Future research should focus on delirium and postoperative complications and clinical care in order to further examine the increased mortality.

## CONFLICT OF INTEREST

None declared.

## AUTHORS' CONTRIBUTION

Jindong Ding Petersen (JDP) wrote the manuscript. Frans Boch Waldorff initiated the project concept. Sonja Wehberg (SW), Volkert Dirk Siersma (VDS), Frans Boch Waldorff (FBW), and JDP discussed and finalized the statistical analysis methods. Sonja Wehberg conducted the statistical analysis. Connie Thurøe Nielsen (CTN) provided her academic knowledge regarding psychiatric and geriatrics care. Bjarke Viberg (BV) provided his expert knowledge regarding hip fracture surgery. All the authors interpreted the study results, commented, and contributed to the manuscript. All authors approved the manuscript for publishing to this journal.

### Peer Review

The peer review history for this article is available at https://publons.com/publon/10.1002/brb3.1823.

## Data Availability

For legal reasons, the data that support the findings of this study cannot be made publicly available. However, with permission, researchers can obtain access to the data from Statistics Denmark.
